# Life Lost

**Published:** 1858-10

**Authors:** 


### LIFE LOST.

THE figure **1** stands over the second entrance from Canal street in Center, in a large, gothic-coped, lead-colored brick building in New York, one block east of Broadway. Just inside of this entrance, to the left, is a narrow winding stairway, leading to the second floor, and passing upward to another floor, any printer, editor, publisher, or man of mind, by turning at right angles to the left again, will, in five or six steps, come bolt up against a new, unpainted, pine-board door, in a wooden partition, made of forty kinds and colors of planks, be the same more or less; on that door is a bit of paper, tacked askew, on which is traced, in lines as straight as a bug's hind-leg: "In from 11 to 2." If the visitor "comes to time," the door will open, in answer to a knock, into an "eight-by-ten" apartment, and at once he will confront one of the most remarkable men and machines of this age or nation. We are thus particular as to locality, to enable our readers from abroad to find the place, and at the proper time, without the labor lost of inquiring of any score of people whom he may meet—for not one in a thousand of our citizens ever heard of the machine, or the man who made it. One of the glorious advantages of a large city is, people are obliged to mind their own business, and pretty diligently, too, or they will soon go to the wall. Not one in a multitude can tell where WILLIAM B. ASTOR lives; and there are tens of thousands in New York, who never heard of HALL'S JOURNAL OF HEALTH." *Mirabile dictu!* By the side of the machine there stands, in his shirt-sleeves, a man, pale, slender, under medium height, forehead broad and towering, hair thrown back, blue eyes, with an expression of countenance kindly, frank, and fearless. At every utterance, those blue eyes twinkle, and a benign smile plays over the whole face. MORSE is showered with praise for a ten years' tug at Telegraphy; FIELD is lauded to the skies for his indomitable perseverance, three years long; and Mr. EVERETT is handsomely paid in the sweeter coin of Beauty's smiles, at the home of his childhood, for a Winter's work, in contriving a successful paying-out apparatus—all stimulated, meanwhile, by knowing that the civilized world was looking on as witnesses, to cheer for success, and for defeat to — hiss!! And what is not, by any means, an inconsiderable item, they lived in plenty, and even in elegance, all this while. No need of working hard to-day, at something else, to secure to-morrow's material of time, and brain, and muscle, to think and work with. But to work and think by turns, by day and night, unnoticed and unknown, and in poverty, and, last, in wasting disease, and for a term of *twenty years!* That is a bravery which beats them all. As to the skill, inventive power—only think of a machine whose distinct parts are counted by thousands, and which, by the turning of a wheel, performs a work, faster, more perfectly, and with greater correctness than is possible for man! All this does TIMOTHY ALDEN'S Printing Machine, as we have seen. It takes type in "pi," or in stick, and with unerring accuracy distributes each of a kind to its own place; and, at the same instant, takes up these types, at the bidding of a human finger, ready for placing them in the press, with a rapidity of three to one at the very least. In a book-publisher's office, one man, with this machine, will do the work of five men, with this additional advantage—it cannot make a mistake in the distribution of "pi," or reading matter; and if the workman spells correctly, it cannot make a mistake in that connection: hence, "reading proof" and corrections are at an end. That a dumb machine should take the type which has just printed a newspaper, and place every A, B, C, &c., exactly in the same receptacle, and never by any possibility make a mistake, from one week's end to another, is incredible to any one, until he sees it; on examination, he will be convinced that a mistake is impossible; and equally so in the resetting, if the operator spells accurately.

But the generous reader will be pained to learn, that the inventor of this wonderful mechanism has so shattered his health, by the intense and protracted application which he has given in its contrivance, that now that it is finished, and the triumph is complete, the joy of it is tarnished by the approach of an insidious disease, which, unless arrested soon, must conquer the conqueror—a fate not uncommon to the sons of genius, who have so often been among the truest benefactors of the race—not that it is a necessary result; but from the simple fact that such men, in their devotion to their cherished object, forget to pay that attention to the preservation of their health, which would not only save it, if given, but would add to its vigor, largely protract the duration of life, and with it would fill up the measure of their triumphs, and gild their setting sun with the golden tints of a prosperous, kindly, and happy old age.

